# Trends in global health financing

**DOI:** 10.1136/bmj.l2185

**Published:** 2019-05-20

**Authors:** Marco Schäferhoff, Sebastian Martinez, Osondu Ogbuoji, Miriam Lewis Sabin, Gavin Yamey

**Affiliations:** 1 Open Consultants, Berlin, Germany; 2 University of Glasgow, Glasgow, UK; 3 Center for Policy Impact in Global Health at Duke University, Durham, NC, USA; 4 Partnership for Maternal, Newborn and Child Health, Geneva, Switzerland; Correspondence to: M Schäferhoff mschaeferhoff@openconsultants.org

## Abstract

Low income countries are still unable to fund a basic package of health services

Three recent reports give us a good picture of global health spending. In December 2018, the World Health Organization updated its global health expenditure database, providing country expenditures up to 2016.^1^ In the same month, the development assistance committee of the Organisation for Economic Cooperation and Development (OECD) released new data on official development assistance for health, which are now available up to 2017.^2^ In January 2019, Policy Cures Research published its latest g-finder survey, tracking global spending on product development for neglected diseases up to 2017.^3^ These three data sources allow us to examine trends in domestic and donor financing for health and assess whether the world is on track to mobilise the financing needed to reach the health targets set out in the third sustainable development goal (SDG 3), which includes achieving universal health coverage.

## Financial obstacles

The Lancet Commission on Investing in Health found that the annual costs of an “essential package” of 218 interventions to achieve universal health coverage would be about $100 (£78; €90) per head, while a more basic package of 108 “highest priority interventions” would cost $50 per head.^4^ How are countries doing in self financing these two packages?

Government spending on health is rising, which is good news, but health spending remains too low in many countries. Expenditure per head roughly doubled in real terms between 2000 and 2016, from $130 to $270 in upper middle income countries and from $30 to $58 in lower middle income countries (based on World Bank income classification in 2016 and unweighted country averages).^5^ Out of the 49 lower middle income countries with 2016 data, nine can afford the essential package of interventions and 16 the more basic package. Twenty four countries can afford neither.^1^

In low income countries, average government spending per head increased from $7 in 2000 to just $9 in 2016. None can afford even the basic package of interventions. Adding in donor funding for health, low income countries still spent only $19 per head in 2016. Furthermore, median out-of-pocket spending on health represents more than 40% of total health expenditures in low income countries. These numbers are a stark reminder of the obstacles to achieving SDG 3.

The new WHO data suggest that governments are not yet making health a high enough priority, as measured by the proportion of all government spending devoted to the health sector (a commonly used metric of prioritisation). The growth in public spending noted above was largely driven by economic growth and fiscal expansion, rather than by giving priority to health. In lower middle income countries, government health spending as a share of general expenditure grew in real terms from only 7.6% in 2000 to 8.3% in 2016. In low income countries, health expenditure fell as a share of government spending, from 7.9% in 2000 to 6.8% in 2016. Indeed, from 2000 to 2016, low income countries became increasingly reliant on official development assistance for health. Increasing OECD assistance may, however, have led governments to reallocate their domestic health spending to other sectors, a phenomenon known as aid fungibility.

**Fig 1:**
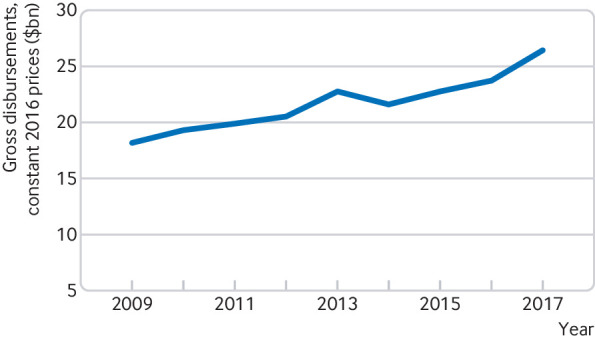
Trend in official development assistance for health.^2^ (including private flows from the Gates Foundation)

OECD figures show that in 2017, official development assistance for health reached $23.9bn, and the Bill and Melinda Gates Foundation, the largest private funder, gave $2.5bn in health aid, giving a combined total of $26.4bn (fig 1). Health aid reached its highest ever level that year. Such aid is again showing a rising trend after a period of stagnation since 2013. The share of official development assistance for health out of total official development assistance increased from 11.7% in 2016 to 12.6% in 2017. A similar upward trend is occurring in funding for product development for neglected diseases, which reached an all time high of $3.6bn in 2017, an increase of 7% ($232m) over the previous year. However, Young et al estimate that about $6bn will be needed annually to advance the current product pipeline.^6^ The funding gap is particularly large for late stage (phase III) clinical trials.

## Mixed picture

What do these three trends mean for global health? The overall picture is mixed. Absolute levels of health spending are rising—but they remain too low in many countries to finance universal health coverage, and health is still not given enough priority by governments. Governments should more strongly prioritise health in their budgets. Over the next few years, over a dozen middle income countries will become ineligible for assistance from funders such as Gavi, the Vaccine Alliance, and many of these countries are vulnerable to disease resurgence.^7^ The positive trend in official development assistance for health and research financing for neglected diseases could be threatened by a looming global economic recession,^8^ and it is essential that donors strongly support upcoming replenishments of global funds.^9^

## Notes

Provenance and peer review: Not commissioned, externally peer reviewed.
